# Supplementary material for: An empirical evaluation of Lex/Yacc and ANTLR parser generation tools
Source: PLoS One. 2022 Mar 3;17(3):e0264326. doi: 10.1371/journal.pone.0264326 (PMC8893623; doi:10.1371/journal.pone.0264326)
Supplement: Attachment — Submitted filename: response-to-reviewers.pdf [file pone.0264326.s001.pdf]

Reviewer #1:

Comment 1: This is a very useful paper. Empirical studies comparing the usability of different software tools are much needed and relatively uncommon. When software tools are compared, the criteria are mostly concerned with performance metrics rather than usability. This paper would be especially useful for faculty who are designing courses that make use of parser generators, but it would also be useful for software engineers in general. The paper is easy to read. There were only a few minor typos. For example, on line 48 "related related" should probably be "related work".

Response 1: Thank you very much for the positive feedback. We have replaced the mistaken "related related" text with "related work".

Comment 2: I did have one minor issue with the paper. The purpose of any course is for students to learn the concepts and to acquire the skill to make use of the concepts. Tools are only a means to the end, not the end itself. Unfortunately, the student performance evaluations (i.e., exam grades) combine evaluation on theory and on lab skill. It would have been useful if these two performance evaluations had been analyzed separately.

Response 2: Following the reviewer's suggestion, we have modified the article to show both the student's final grades and the marks they achieve in the lab exam. To this aim, we have modified Figure 4 to include the marks of the lab exam. Now, we can compare, together with the final marks, the lab marks of the previous years and contrast them with the Lex/Yacc and ANTLR group. Interestingly, the lab marks of the ANTLR group are significantly higher than all the previous years, whereas the final marks are only significantly higher than 5 out of the 7 courses.

This is the new Figure 4:

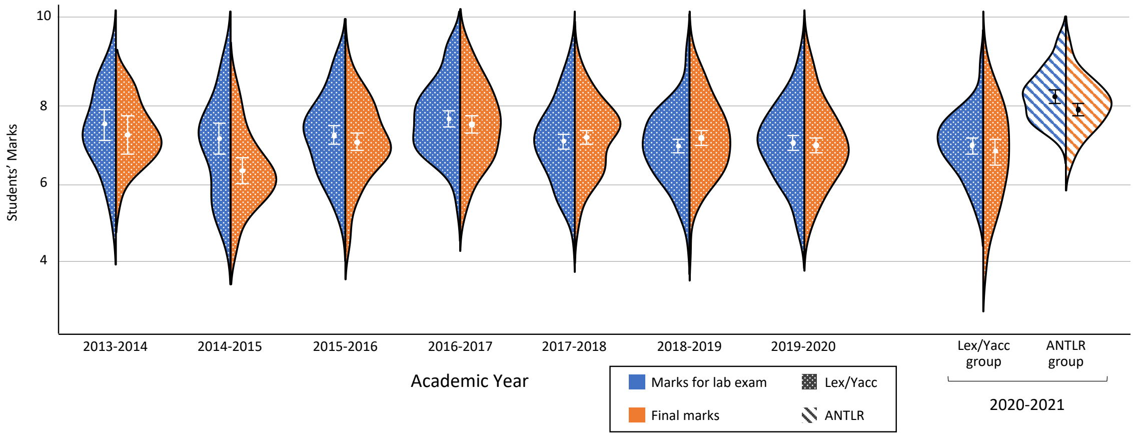

Fig 4. Final and lab marks of the last 8 academic courses. Whiskers represent 95% confidence intervals and the points denote their mean values.

We have changed the article accordingly. This is one paragraph where we describe the results:

> *Fig. 4 presents the same comparison with the previous years, but considering the lab exams and the students' final marks. In the last academic year, the students of the ANTLR group obtained significantly higher marks than those in the Lex/Yacc group (95% confidence intervals do not overlap) for both the lab exams and the final marks. Compared to the previous years, Lex/Yacc group shows no difference for both kinds of assessments. However, ANTLR has significantly higher values than the rest of the courses for the lab exam, and for all of them but two (2013-14 and 2016-17) in the*

case of the final marks. The average lab and final marks of the students of the ANTLR group are the highest ones among all the years.

The ANTLR group obtained better final marks, but that was because the students obtained significantly better performance in the lab exam (theory and lab exams are worth 50% each). There were no significant differences in the theory exams when ANTLR was used. This means that ANTLR seems to facilitate the implementation of a language (lab exam), but it does not seem to provide a significantly better understanding of the theoretical concepts (theory exam), compared to Lex/Yacc.

We have modified the discussion section to include this:

... This leads us to think that ANTLR eases the implementation of the programming language. It might also help to understand the theoretical concepts, but there were no significant differences in the theory marks (the higher final marks of the ANTLR group were caused by the increase in their lab marks).

Reviewer #2:

Comment 1: This research is of interest to researchers in language design and to instructors in software engineering and computer science. The specific question examined is fundamental to many aspects of practical programming.
Generally the work meets best practice in empirical software engineering, however there could be improvements to the way it is written up. I recommend use of the guidelines in: http://people.ucalgary.ca/~far/Lectures/SENG421/PDF/Guidelines.pdf

Response 1: We thank the reviewer for his/her kind words and the reference to the guidelines for empirical research in software engineering. Following his/her advice, we have used the guidelines to improve the article. For each guideline, we have analyzed to what extent we fulfill it. When necessary, we indicate how we have changed the article to meet each guideline or to indicate a limitation of our study.

This is a summary of the analysis undertaken:

- Topic C: Experimental context.
  - C1: *Be sure to specify as much of the industrial context as possible. In particular, clearly define the entities, attributes, and measures that are capturing the contextual information*. We have extended the description of the context of the course under study to clarify the expected skills of the students enrolled in our course. All the following text but the first sentence has been added to the Context section:

    We conducted an experiment with undergraduate students of a Programming Language Design and Implementation course [25] in a Software Engineering degree [26], at the University of Oviedo (Spain). The course is delivered in the second semester of the third year. The students have previously attended five programming courses, three of them in Java, and the other two in C# and Python. Such courses cover different topics such as procedural, object-oriented, functional, and concurrent programming [27], and human-computer interaction [26]. They have also taken other courses that include Java programming assignments such as software design, algorithmics, data structures, web development, databases, numeric computation, distributed systems, information repositories, computer architecture, and operating systems [26]. In four of these courses, the students have to implement a real Java application following a project-based learning approach.

  - C2: *If a specific hypothesis is being tested, state it clearly prior to performing the study and discuss the theory from which it is derived, so that its implications are apparent.* This guideline is already met in the article, where we perform different hypothesis tests. As an example, this text is taken from the Evaluation section:

    … We want to see if there are statistically significant differences between the values of the two groups of students. Since the two groups are independent and students' marks are normally distributed (Shapiro-Wilk test [35], p-value>0.1 for all the distributions), we apply an unpaired two-tailed t-test (α=0.05) [36]—the null hypothesis (H0) states that there is no significant difference between the means of the two groups.

  - C3: *If the research is exploratory, state clearly and, prior to data analysis, what questions the investigation is intended to address and how it will address them*. All the research questions were stated before designing the experiments.

  - C4: *Describe research that is similar to, or has a bearing on, the current research and how current work relates to it.* We describe that in the Related Work section.

- Topic D: Experimental design
  - D1: *Identify the population from which the subjects and objects are drawn*. In the previous version of the article, we only identified the number and gender of students. Now, we include additional information such as the average and standard deviation of the age of the students. We also indicate that students retaking the course were not considered in the study, because their previous knowledge might bias the results. This is the new text in the Context section:

    ...The 183 students (131 males and 52 females) enrolled in the course were divided into two random groups: the first group had 92 students (67 males, 25 females, average age 22.8, and standard deviation 2.04) and the second one was 91 (64 males, 27 females, average age 22.7, and standard deviation 2.13). Retakers were not included in our study, because they have previous knowledge about the course and experience in the utilization of Lex/Yacc (the tool used in the previous years).

  - D2: *Define the process by which the subjects and objects were selected*. We explicitly indicate the process by which the students were selected (Context section):

    Our study took place in the second semester of the 2020-2021 academic year. The 183 students (131 males and 52 females) enrolled in the course were divided into two random groups: the first group had 92 students (67 males, 25 females, average age 22.8, and standard deviation 2.04) and the second one was 91 (64 males, 27 females, average age 22.7, and standard deviation 2.13)...

  - D3: *Define the process by which subjects and objects are assigned to treatments*. The main text that describes such process is (Context section):

    For the first part of the course (the first seven weeks), lectures and labs for the first group were delivered using BYaccJ/JFlex (Java versions of the classic Yacc/Lex generators). For the second group, ANTLR was used instead. The second part of the course has the same contents for both groups, since there is no dependency on any generation tool. Both groups implemented the very same programming language, and they had the same theory and lab exams.

  - D4: *Restrict yourself to simple study designs or, at least, to designs that are fully analyzed in the statistical literature.* Our study design is based on simple methods fully analyzed in the statistical literature and cited accordingly in the article.
  - D5: *Define the experimental unit.* In our work, the experimental unit is the student. We have modified the first sentence in the Evaluation section to state that:

    We analyze the influence of Lex/Yacc and ANTLR generators in students' performance (students are the experimental units) with different methods...

  - D6: *For formal experiments, perform a pre-experiment or precalculation to identify or estimate the minimum required sample size*. As suggested by this guideline, we have computed the power of the hypothesis tests to the new version of the article. Those values have been added as a new column to Table 6 ($1-\beta$). All the values obtained are greater than 0.8, the threshold commonly used as a standard for adequacy [38]. This is the new text added to the Results section:

    ... Table 6 also presents the power of the tests. The power of a hypothesis test is the probability that the test correctly rejects the null hypothesis, denoted by $1-\beta$ (β represents the probability of making a type II error) [38]. For all the tests, we obtain values above 0.8, which is the threshold commonly taken as a standard for adequacy for the given sample sizes [38]. Therefore, we reject the null hypothesis and hence conclude that there are significant differences between the two groups...

- D7: *Use appropriate levels of blinding*. In our study, the students knew the parser generation tool they were using. They could have spoken to one another and caused expectations about the utilization of a different tool (ANTLR), not used in the previous years. Thus, we have added a new discussion about this topic in the Threats to Validity section. This is the new paragraph:

  In an empirical research study where users utilize two different tools, the participants are aware of the tool they are using, so a blind experiment is hardly feasible [24]. Therefore, the student's expectations about the use of ANTLR may have represented a psychological factor that might have influenced their opinion of that tool. Although the lecturers did not compare ANTLR with Lex/Yacc, the students may have known they were using different tools because they could have spoken one to another. However, the measurements that are not based on student's opinion (completion percentages, work time, and evaluation data) seem to back up the answers that the students gave in the questionnaires.

- D8: *If you cannot avoid evaluating your own work, then make explicit any vested interests (including your sources of support) and report what you have done to minimize bias*. This guideline is not applicable in our study, since neither ANTLR nor Lex/Yacc are our own work.

- D9: *Avoid the use of controls unless you are sure the control situation can be unambiguously defined*. Our work compares two well-known parser generation tools, one against the other.

- D10: *Fully define all treatments (interventions)*. The article describes the treatments in two main parts. First, Table 3 depicts the work to be done by the students in each lab. Then, the features of the language to be implemented are described in the following text:

  … Labs (Table 3) follow a project-based learning approach, and they are mainly aimed at implementing a compiler of a medium-complexity imperative programming language. That language provides integer, real and character built-in types, arrays, structs, functions, global and local variables, arithmetical, logical and comparison expressions, literals, identifiers, and conditional and iterative control flow statements [25]. The compiler is implemented in the Java programming language.

- D11: *Justify the choice of outcome measures in terms of their relevance to the objectives of the empirical study.* The Evaluation section in the paper justifies the choice of measures in terms of the objectives of our study. First, we indicate we want to analyze the influence of the tools in students' performance:

  We analyze the influence of Lex/Yacc and ANTLR generators in students' performance (students are the experimental units) with different methods…

  Then, we describe the different methods to measure their performance: calculating the completion levels for each lab; recording the number of additional autonomous hours to finish students' work; processing their grades in the lab and theory exams; and comparing their marks with previous years.

  We also ask for their opinion about the tool used, after each lab:

  To gather the student's opinion about the lexer and parser generators used, we asked them to fill in the anonymous questionnaire shown in Table 4, published online with Google Forms. They filled in the questionnaire after the labs where the generators

were first used (labs 4 and 5), and after implementing the first part of the language (lab 7) and the whole compiler (lab 15). Questions 4 and 5 were only asked in lab 15, since students had yet not implemented the semantic analysis and code generation modules in labs 4, 5, and 7…

- Topic DC: Conduct of the experiment and data collection.

  - DC1: *Define all software measures fully, including the entity, attribute, unit and counting rules.* One of the measures that we had not defined clearly in the previous version of the article is the completion level of students' work after each lab. Thus, we have added the following text to better describe how we compute that percentage:

    … First, we measure the completion level of students' work after each lab focused on the use of lexer and parser generators (i.e., labs 4 to 6 in Table 3). Such completion level is measured with a percentage indicating to what extent all the functional requirements of each lab have been met by the student. To this aim, we define a rubric and the lab instructor annotates the completion percentage for each student, after the lab. We perform the same analysis when we review their work after the first part of the course (lab 7) and for the whole compiler (lab 15).

  - DC2: *For subjective measures, present a measure of interrater agreement, such as the kappa statistic or the intraclass correlation coefficient for continuous measures.* We had not included any measure of inter-rater agreement in our first article, so we have included it in the new version. Since we need to measure the inter-rater agreement of multiple students, the Cohen's kappa statistic cannot be used. Thus, we have measured the Krippendorff's alpha coefficient for ordinal data (Likert scale) for all the questionnaires. This is the new text that discusses the results (Evaluation section):

    … Likewise, we use the Krippendorff's α coefficient for ordinal data to measure inter-rater reliability [33]. Substantial reliability (α>0.8) is obtained for all the questionnaires but lab 15 for Lex/Yacc, which shows modest reliability (α=0.781) [34].

  - DC3: *Describe any quality control method used to ensure completeness and accuracy of data collection.* We perform the following quality control methods: for all the measurements, we identify and treat outliers (see guideline A3), we compute the Cronbach's α coefficient to measure the internal consistency of our questionnaires (Evaluation section), and we check for normality when t-tests are applied (Evaluation).

  - DC4: *For surveys, monitor and report the response rate and discuss the representativeness of the responses and the impact of nonresponse.* We had not stated the exact number of students attending each lab and answering each questionnaire. Therefore, we have extended the new version of the article to include that information. Table 5 now indicates the number of students attending the labs where we perform measurements. Likewise, a new column has been added to Table 7, indicating the number of students that answered the questionnaire. The greatest difference between the number of students in a group and those attending the labs or filling in the survey is 7.6% (Lex/Yacc group, questionnaire of the last lab). For the initial labs, almost 100% of the students attended the labs and filled in the questionnaire. The lowest response rates were obtained in the last lab (4.3% for attendance and 7.6% for the survey), and the main reason is that those students had quit the course.

  - DC5: *For observational studies and experiments, record data about subjects who drop out from the studies.* The students who dropped out from the course were 4.3% for the Lex/Yacc group and 2.2% for ANTLR. These low percentages do not seem to

represent an impact on the results, since all the values obtained for the power of the tests with those sizes were greater than 0.8 (see guideline D6). Moreover, the lab work is individual, so dropping-out students do not affect the work of the others.

- DC6: *For observational studies and experiments, record data about other performance measures that may be affected by the treatment, even if they are not the main focus of the study.* We do not identify any other performance measures that may be affected by our study. The main variable is students' performance and it is analyzed in the article.

- Topic A: Analysis.
  - A1: *Specify any procedures used to control for multiple testing*. We do not undertake multiple testing in our work.
  - A2: *Consider using blind analysis*. All the analyses done are aimed at analyzing the differences between two groups, so blind analysis is not applicable.
  - A3: *Perform sensitivity analyses*. We searched for outliers in the numerical values of completion rates (Tables 5 and 6) and the number of additional hours to finish the labs (Table 6). All the values were within the Q_3-1.5*IQR and Q_3+1.5*IQR (Tukey's rule, k=1.5). It is worth noting that we did not consider the students who retook the course. This piece of information was not originally in our article, so we have added it to the Context section:

    … Retakers were not included in our study, because they have previous knowledge about the course and experience in the utilization of Lex/Yacc (the tool used in the previous years).
  - A4: *Ensure that the data do not violate the assumptions of the tests used on them*. To apply all the t-tests we performed in our analysis, we checked that it follows a normal distribution computing the Shapiro-Wilk test. In all the tests, p-values were greater than 0.01. This is the text in the article that mentions that:

    … Since the two groups are independent and students' marks are normally distributed (Shapiro-Wilk test [35], p-value>0.1 for all the distributions), we apply an unpaired two-tailed t-test (α=0.05) [36]…
  - A5: *Apply appropriate quality control procedures to verify your results*. We undertook exploratory data analyses and visualization before undertaking our analyses.

- Topic P: Presentation of results.
  - P1: *Describe or cite a reference for all statistical procedures used*. All the statistical procedures we used have been cited. Some of them have also been briefly described. In our new version of the article, we have added references to the Shapiro-Wilk normality test and to the Student's t-test, which had not been included originally.
  - P2: *Report the statistical package used*. We had not reported that in the article, so the following sentence has been included at the end of the Methods section:

    All the statistical computations were performed with IBM SPSS Statistics 27.0.1…
  - P3: *Present quantitative results as well as significance levels. Quantitative results should show the magnitude of effects and the confidence limits*. The only quantitative result in the guideline that we had not included is the t statistic. Thus, we have added a new column to Table 6 to include the t statistic of the t-tests.
  - P4: *Present the raw data whenever possible. Otherwise, confirm that they are available for confidential review by the reviewers and independent auditors*. We have taken the raw data gathered from all the experiments described in the article and uploaded

them to [37]. The following text has been added to the last paragraph of the Evaluation section:

> *... The raw data obtained from conducting all the experiments are available for download from [37].*

- P5: *Provide appropriate descriptive statistics*. This guideline was followed in the interpretation of student's opinion. As described by Boone *et al.* [39] the Likert scale data obtained by summing the values of four or more Likert-type items can be used to compare values at the interval measurement scale [39]. That is precisely what we do in Figure 2: we computed the combined (sum) Likert scale values for Q1-Q5 and then perform t-test to compare the two groups.
- P6: *Make appropriate use of graphics*. All the guidelines for graphical representations described in P6 have been followed. We have also respected the principles of "The Visual Display of Quantitative Information" by Edward Tufte.

- Topic I: Interpretation of results.
  - I1: *Define the population to which inferential statistics and predictive models apply*. We have thoroughly described the population (guideline D1) and its context (guideline C1). Regarding the distribution of the attribute values of the population, we have first checked for their normality before applying the statistical tests.
  - I2: *Differentiate between statistical significance and practical importance*. For all the hypothesis tests undertaken to compare the values of the ANTLR and Lex/Yacc groups, we compute the p-value as a measure of significance. Afterwards, when a significance level is achieved, we calculate the difference between the mean values of both distributions and discuss the practical importance of that difference.
  - I3: *Define the type of study*. This guideline cites the study of Zelkowitz and Wallace [42] as a valid reference for classifying empirical research work in software engineering, where 12 types of experimental methods are identified. According to their study, our work is a controlled experiment within a synthetic environment. We now define the type of study in the following text included in the Threats to Validity section:

    > *Likewise, the study was conducted with Software Engineering students with Java programming skills, all of them beginners of the parser generation tools (students retaking the course were not considered). According to Zelkowitz and Wallace, this kind of validation method is a controlled experiment within a synthetic environment, where two technologies are compared in a laboratory setting [42]...*

  - I4: *Specify any limitations of the study*. We have the Threats to Validity section in the article to specify all the limitations of our study.

Finally, we have included a reference to the guidelines pointed out by the reviewer at the beginning of the Methods section:

> *... We followed the guidelines by Kitchenham et al. for empirical research works in software engineering [24].*

Reviewer #3:

Comment 1: I'm glad to see research being done to help justify the move from established software to newer (and arguably better) alternatives. Overall, I don't find any major issues with this paper, however, a few recommendations that I'd like to see prior to its publishing:

1) I would suggest moving the related work section up towards the front of the paper and used to help motivate why ANTLR is being considered in this study.

Response 1: We have followed the reviewer's suggestion and moved the Related Work section. In the new version of the article, the related work is presented just after the introduction.

Comment 2: 2) In the results section, I would like to see plots of the actual distribution of the data, especially if it can be overlapped to clearly show how different each group is, and the percentage of data that lies outside the distribution of the opposing group.

Response 2: We have modified Figures 2 and 4 to include the actual distribution of the data. We have replaced the bar charts with violin plots that include the distribution of the data. Each violin is divided into two parts to compare two distributions. Inside each part of the violin, 95% confidence intervals are drawn. If they do not overlap, it means there is a statistically significant difference between both distributions [40].

This is the new Figure 2, where the combined Likert scale values for questions Q1 to Q5 are compared for the Lex/Yacc and ANTLR groups:

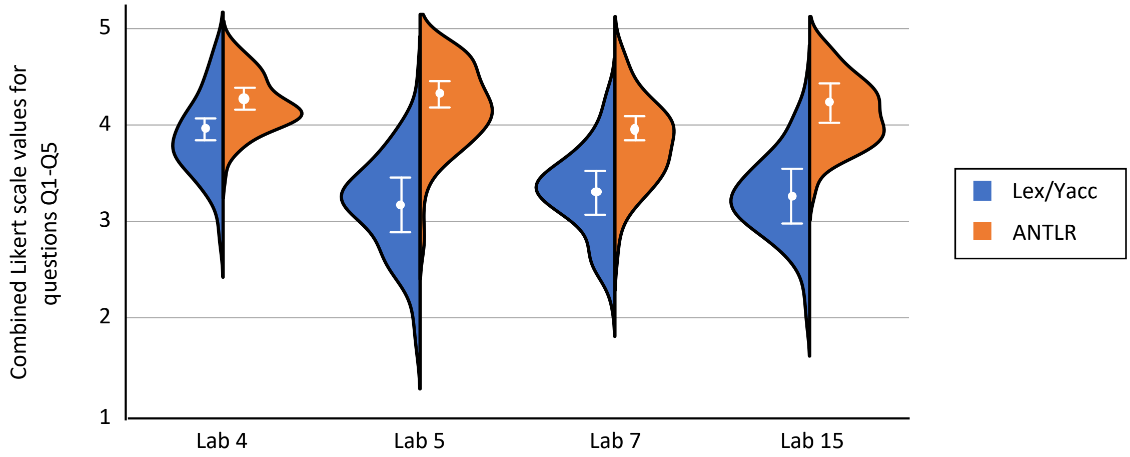

Fig 2. Combined Likert scales for the questions Q1-Q5 of the questionnaire in Table 5. Whiskers represent 95% confidence intervals and the points denote their mean values.

Likewise, the new Figure 4 uses the same approach to compare the lab and final marks of the last 7 years and the one under study. The color of the distribution represents either the lab or the final marks of the students, whereas the pattern represents the parser generation tool (Lex/Yacc or ANTLR):

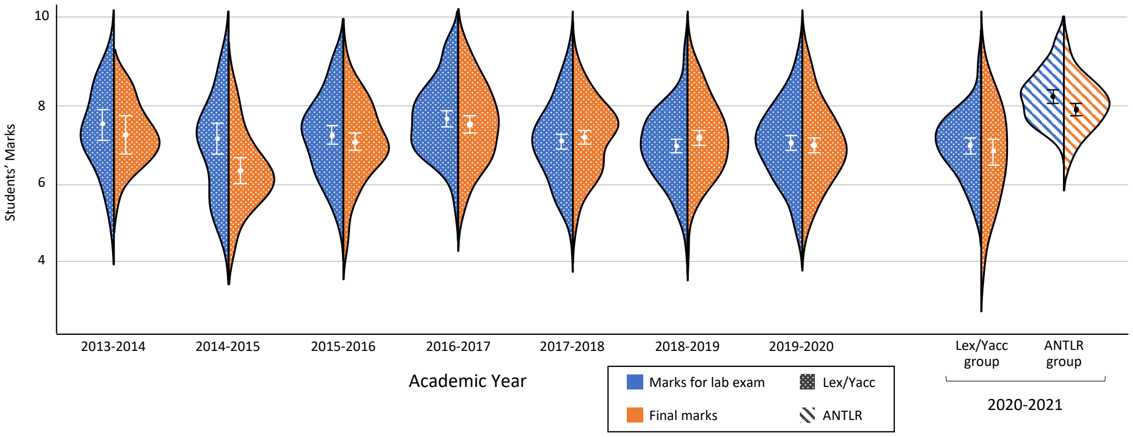

Fig 4. Final and lab marks of the last 8 academic courses. Whiskers represent 95% confidence intervals and the points denote their mean values.

Moreover, if the reader would like to check the raw data produced in all the experiments, we have uploaded them to a website and included a reference to it in the article:

> *... The raw data obtained from conducting all the experiments are available for download from [37].*

Comment 3: Furthermore, I think that there should be a better explanation of what exactly "1% work completed" means. How does that translate into non-percentage units? Are we talking 1 additional function in the assignment? 10 lines of code? 1 step of a 20 step process? Including the actual numbers alongside the percent would help to make the results a little more useful. If ANTLER allows students to get 10 more lines of code written during a lab, I might question whether or not its worth converting my entire course over to using it - but if 10 more students complete the assignment using ANTLR over Lex/Yacc, then its absolutely worth the switch.

Response 3: We apologize for the lack of clarity in our explanation. Such percentage measures to what extent the student has managed to meet all the functional requirements in each lab. For example, in the lexical analysis lab (lab 4), they have to implement a collection of lexical patterns for the given imperative language. We, the lecturers, define a rubric where completion levels (the requirements met) are associated with percentage values (e.g., 100% means all the lexical patterns have been correctly specified with the tool). After each lab, the instructor annotates each student's work using the rubric. The number obtained is the completion percentage we mention in the article.

We have changed the paper to clarify the issue detected by the reviewer. This is the new text (Evaluation section):

> *... First, we measure the completion level of students' work after each lab focused on the use of lexer and parser generators (i.e., labs 4 to 6 in Table 3). Such completion level is measured with a percentage indicating to what extent all the functional requirements of each lab have been met by the student. To this aim, we define a rubric and the lab instructor annotates the completion percentage for each student, after the lab...*

We thank the reviewer for his/her comments to help us improve the quality of the paper.

Reviewer #4:

Comment 1: This paper shows an empirical study on the performance of two groups of undergraduate students of software engineering implementing a compiler using Lex/Yacc and ANTLR, respectively. The authors measure students' completion rates and time spent for labs, attendance and pass/fail rates for exams, and opinions regarding the use of the tools. All data are shown in tables and figures, indicating the superiority of ANTLR over Lex/Yacc in teaching and helping students learn compiler construction more efficiently.

Overall, I think this paper is very interesting and is focused on an important issue for educators. The authors have conducted extensive data collection and quantitative analysis to support the conclusion. One interesting finding in the paper is that the choice of tools for programming assignments affects students' performance in paper exams. A shallow cause could be that students using ANTLR had more hands-on experiences, which allow them to understand the theoretical parts better. Nevertheless, a deeper one probably involves some psychological factors, which I would appreciate if the authors could include some.

Response 1: We thank the reviewer for his/her kind words about the article. In his/her comment, the reviewer mention two important topics about how the choice of lexer/parser tools affects students' performance. First, the reviewer mentions that a shallow cause could be that students using ANTLR had more hands-on experiences. Regarding their experience before the course, we did not consider students who retake the course in our study, because that would bias the comparison between the tools. We have also analyzed their ages to see if there is a significant difference between both groups, but their distributions overlap. To clarify this, we have included this information in the Context section. This is the modified text:

> *… The 183 students (131 males and 52 females) enrolled in the course were divided into two random groups: the first group had 92 students (67 males, 25 females, average age 22.8, and standard deviation 2.04) and the second one was 91 (64 males, 27 females, average age 22.7, and standard deviation 2.13). Retakers were not included in our study, because they have previous knowledge about the course and experience in the utilization of Lex/Yacc (the tool used in the previous years).*

Likewise, to clarify that the contexts of students are the same, we have added the following paragraph at the beginning of the same section:

> *We conducted an experiment with undergraduate students of a Programming Language Design and Implementation course [25] in a Software Engineering degree [26], at the University of Oviedo (Spain). The course is delivered in the second semester of the third year. The students have previously attended five programming courses, three of them in Java, and the other two in C# and Python. Such courses cover different topics such as procedural, object-oriented, functional, and concurrent programming [27], and human-computer interaction [26]. They have also taken other courses that include Java programming assignments such as software design, algorithmics, data structures, web development, databases, numeric computation, distributed systems, information repositories, computer architecture, and operating systems [26]. In four of these courses, the students have to implement a real Java application following a project-based learning approach.*

All these data do not seem to imply that students using ANTLR had more hands-on experiences.

Regarding the connection between the use of ANTLR and the better understanding of the theoretical parts, we have analyzed the relation between the theory marks and the use of different parser generation tools. The results showed that there are no significant differences in the theory exams when ANTLR was used. The ANTLR group obtained better final marks, but that was because the students obtained significantly better performance in

the lab exam (theory and lab exams are worth 50% each). This means that ANTLR seems to facilitate the implementation of a language (lab exam), but it does not seem to provide a significantly better understanding of the theoretical concepts (theory exam), compared to Lex/Yacc. We have included the following new text in the Discussion section to clarify that:

> *... This leads us to think that ANTLR eases the implementation of the programming language. It might also help to understand the theoretical concepts, but there were no significant differences in the theory marks (the higher final marks of the ANTLR group were caused by the increase in their lab marks).*

The second topic mentioned by the reviewer is related to the psychological factors that might have influenced our study. According to the guidelines described by Kitchenham *et al.* about how to do empirical research in software engineering [24], it is true that there might be a psychological factor caused by the fact that the students know they are using different tools. If they know ANTLR has more advanced features than Lex/Yacc, it could influence the answers to the questionnaire.

We have modified the article to include the topic pointed out by the reviewer. The following new discussion has been added to the Threads to Validity section:

> *In an empirical research study where users utilize two different tools, the participants are aware of the tool they are using, so a blind experiment is hardly feasible [24]. Therefore, the student's expectations about the use of ANTLR may have represented a psychological factor that might have influenced their opinion of that tool. Although the lecturers did not compare ANTLR with Lex/Yacc, the students may have known they were using different tools because they could have spoken one to another. However, the measurements that are not based on student's opinion (completion percentages, work time, and evaluation data) seem to back up the answers that the students gave in the questionnaires.*

Comment 2: Another complaint is that all figures are not in place but at the end of the manuscript, causing the reading experience to be a little unpleasant.

Response 2: We apologize for that, but it is a requirement of the journal.

I quote from the journal guidelines (https://journals.plos.org/plosone/s/submission-guidelines):

> *Figures*
>
> *Do not include figures in the main manuscript file. Each figure must be prepared and submitted as an individual file.*
>
> *Cite figures in ascending numeric order at first appearance in the manuscript file.*

For some reason, they ask the authors to place the figures at the end of the manuscript. Later, when the article is published, they include the figures inside the text.

We would like to thank the anonymous reviewer for his/her suggestions and comments.

Reviewer #5:

Comment 1:

In this manuscript, the authors conduct an empirical study to compare two widely used parser generation tools: ANTLR and Lex/Yacc. Specifically, they design experiments to evaluate the two tools from the following aspects: language implementor productivity, simplicity, intuitiveness, and maintainability. The experiment results demonstrate that ANTLR yields better performance based on the measured features.

From my point of view, the contribution of this work is twofold:

1. The authors design several metrics to measure the two parser generation tools. In particular, the metrics are defined based on the completion level of students' work, additional time that the students needed to finish the labs. Also, students' options on the tools (through questionnaire) are collected for the analysis of the tools. Those metrics (features) can help us better understand the tools.

2. The experiments are conducted in "large" scale since there are more than 90 students in each group. Also, the authors provide statistical significance evidences when comparing the differences of the two parser generation tools.

At the same time, I have two concerns about this work:

1. All the experiments are based on students from the "Programming Language Design and Implementation" course, and those students are all beginners of the tools (this is my assumption). In my opinion, we can not say one tool is better than the other tool only based on the beginners' experience.

Response 1: We agree it could not be said that one tool is better than the other one by conducting an empirical evaluation with students. The first measure we have taken to avoid such a claim is modifying different parts of the article to make sure that we do not state that in the text. First, the last sentence in the abstract has been rewritten to:

> *… Under the context of the academic study conducted, ANTLR has shown significant differences for most of the empirical features measured.*

We have also modified the last sentence in the second last paragraph of the Introduction, where we describe our contribution.

> *… These data provide evidence, under the context of our study, to undertake an empirical comparison of the two parser generators.*

The first sentence of the Conclusions section has also been changed for the same purpose:

> *The empirical comparison undertaken shows that, for the implementation of a programming language of medium complexity by year-3 students of a Software Engineering degree, the ANTLR tool shows significant benefits compared to Lex/Yacc…*

Regarding the beginner level of students with respect to the tool, we have included a paragraph in the Threats to Validity section to indicate that the results should not be generalized to state that one tool is better than the other one. This is the text of that new paragraph:

> *Likewise, the study was conducted with Software Engineering students with Java programming skills, all of them beginners of the parser generation tools (students retaking the course were not considered). According to Zelkowitz and Wallace, this kind of validation method is a controlled experiment within a synthetic environment,*

where two technologies are compared in a laboratory setting [42]. Due to the validation method and the type of user used, the results should not be generalized to state that one tool is better than another one.

Finally, to clarify the level of students involved in our study, we have extended the Context subsection in Methods. Now, the reader will have more information regarding the expected skills of the students involved in our study. This is the new text:

... The course is delivered in the second semester of the third year. The students have previously attended five programming courses, three of them in Java, and the other two in C# and Python. Such courses cover different topics such as procedural, object-oriented, functional, and concurrent programming [27], and human-computer interaction [26]. They have also taken other courses that include Java programming assignments such as software design, algorithmics, data structures, web development, databases, numeric computation, distributed systems, information repositories, computer architecture, and operating systems [26]. In four of these courses, the students have to implement a real Java application following a project-based learning approach.

Comment 2: 2. When measuring the simplicity, intuitiveness, and maintainability of the tools, only one question is used to measure one aspect of the tool. For example, "Q1: I have found it easy to use the tool(s) to generate the lexical and syntactic analyzers" is used to measure the simplicity of the tool. First, I feel the question design is too general/broad, so I am not sure the answers from students are accurately measuring the specific aspect of the tool. Second, I am not sure if the students are measuring the tool in the same scale (especially when the students are not trained on how to assign the scale of the simplicity,) since the concept of simplicity (also for the other features) is different for different students. I am not convinced by the questionnaire design.

Response 2: We have consulted the bibliography regarding the comment about the scale used by the students to measure subjective measures such as simplicity, intuitiveness, and maintainability. In the guidelines for empirical research in software engineering proposed by Kitchenham *et al.* [24], the guideline DC2 indicates: "*For subjective measures, present a measure of interrater agreement, such as the kappa statistic or the intraclass correlation coefficient for continuous measures*." The inter-rater agreement measures the degree of agreement among observers (the students) who rate something (the parser generation tools). Since we need to measure the inter-rater agreement of multiple students, the Cohen's kappa statistic cannot be used. Thus, we have measured the Krippendorff's alpha coefficient for ordinal data (Likert scale) for all the questionnaires. The results showed that there is substantial reliability ($\alpha>0.8$) for all the surveys but one that obtained modest reliability ($\alpha=0.781$).

We have included this information with the following new text in the Evaluation section:

... Likewise, we use the Krippendorff's α coefficient for ordinal data to measure inter-rater reliability [33]. Substantial reliability ($\alpha>0.8$) is obtained for all the questionnaires but lab 15 for Lex/Yacc, which shows modest reliability ($\alpha=0.781$) [34].

The reason why we just asked one question to measure one aspect of the tool is to prevent the students from not filling in the survey. In our experience, that often occurs when we ask them many questions. Moreover, they were asked to fill in four different questionnaires throughout the course.

Regarding the general text of the questions, we had not explained that sufficiently clear in the article. In the secondary caption of Table 4, we indicated "Questions were adapted

depending on the lab delivered" meaning that the text in Table 4 is a generalization of the actual question. For example, in lab 4, the first question was "I have found it easy to use ANTLR to generate the lexical analyzer". To clarify that, we have added this explanation to that caption:

> *Answers are in a 5-point Likert scale, ranging from 1="completely disagree" to 5="completely agree". Questions were adapted depending on the lab delivered (e.g., for lab 4, the exact question for the Lex/Yacc group was "I have found it easy to use Lex to generate the lexical analyzer"). Thus, Q1 to Q3 only ask about lexical analysis in lab 4; for lab 5, they only ask about syntax analysis; and labs 7 and 15 include both analyses.*

To improve our article, we have examined all the guidelines for empirical research in software engineering works described in [24]. That document details 40 guidelines under six different topics. For each guideline, we have analyzed to what extent we fulfill it. When necessary, we indicate how we have modified our paper to meet each guideline or to indicate a limitation of our study. What follows is a summary of the analysis undertaken for the new version of the article:

- Topic C: Experimental context.
  - C1: *Be sure to specify as much of the industrial context as possible. In particular, clearly define the entities, attributes, and measures that are capturing the contextual information*. We have extended the description of the context of the course under study to clarify the expected skills of the students enrolled in our course. All the following text but the first sentence has been added to the Context section:

    > *We conducted an experiment with undergraduate students of a Programming Language Design and Implementation course [25] in a Software Engineering degree [26], at the University of Oviedo (Spain). The course is delivered in the second semester of the third year. The students have previously attended five programming courses, three of them in Java, and the other two in C# and Python. Such courses cover different topics such as procedural, object-oriented, functional, and concurrent programming [27], and human-computer interaction [26]. They have also taken other courses that include Java programming assignments such as software design, algorithmics, data structures, web development, databases, numeric computation, distributed systems, information repositories, computer architecture, and operating systems [26]. In four of these courses, the students have to implement a real Java application following a project-based learning approach.*

  - C2: *If a specific hypothesis is being tested, state it clearly prior to performing the study and discuss the theory from which it is derived, so that its implications are apparent*. This guideline is already met in the article, where we perform different hypothesis tests. As an example, this text is taken from the Evaluation section:

    > *… We want to see if there are statistically significant differences between the values of the two groups of students. Since the two groups are independent and students' marks are normally distributed (Shapiro-Wilk test [35], p-value>0.1 for all the distributions), we apply an unpaired two-tailed t-test (α=0.05) [36]—the null hypothesis (H0) states that there is no significant difference between the means of the two groups.*

  - C3: *If the research is exploratory, state clearly and, prior to data analysis, what questions the investigation is intended to address and how it will address them*. All the research questions were stated before designing the experiments.
  - C4: *Describe research that is similar to, or has a bearing on, the current research and how current work relates to it.* We describe that in the Related Work section.

- Topic D: Experimental design

- D1: *Identify the population from which the subjects and objects are drawn*. In the previous version of the article, we only identified the number and gender of students. Now, we include additional information such as the average and standard deviation of the age of the students. We also indicate that students retaking the course were not considered in the study, because their previous knowledge might bias the results. This is the new text in the Context section:

  ...The 183 students (131 males and 52 females) enrolled in the course were divided into two random groups: the first group had 92 students (67 males, 25 females, average age 22.8, and standard deviation 2.04) and the second one was 91 (64 males, 27 females, average age 22.7, and standard deviation 2.13). Retakers were not included in our study, because they have previous knowledge about the course and experience in the utilization of Lex/Yacc (the tool used in the previous years).

- D2: *Define the process by which the subjects and objects were selected*. We explicitly indicate the process by which the students were selected (Context section):

  Our study took place in the second semester of the 2020-2021 academic year. The 183 students (131 males and 52 females) enrolled in the course were divided into two random groups: the first group had 92 students (67 males, 25 females, average age 22.8, and standard deviation 2.04) and the second one was 91 (64 males, 27 females, average age 22.7, and standard deviation 2.13)...

- D3: *Define the process by which subjects and objects are assigned to treatments*. The main text that describes such process is (Context section):

  For the first part of the course (the first seven weeks), lectures and labs for the first group were delivered using BYaccJ/JFlex (Java versions of the classic Yacc/Lex generators). For the second group, ANTLR was used instead. The second part of the course has the same contents for both groups, since there is no dependency on any generation tool. Both groups implemented the very same programming language, and they had the same theory and lab exams.

- D4: *Restrict yourself to simple study designs or, at least, to designs that are fully analyzed in the statistical literature.* Our study design is based on simple methods fully analyzed in the statistical literature and cited accordingly in the article.

- D5: *Define the experimental unit.* In our work, the experimental unit is the student. We have modified the first sentence in the Evaluation section to state that:

  We analyze the influence of Lex/Yacc and ANTLR generators in students' performance (students are the experimental units) with different methods...

- D6: *For formal experiments, perform a pre-experiment or precalculation to identify or estimate the minimum required sample size*. As suggested by this guideline, we have computed the power of the hypothesis tests to the new version of the article. Those values have been added as a new column to Table 6 (1-β). All the values obtained are greater than 0.8, the threshold commonly used as a standard for adequacy [38]. This is the new text added to the Results section:

  ... Table 6 also presents the power of the tests. The power of a hypothesis test is the probability that the test correctly rejects the null hypothesis, denoted by 1-β (β represents the probability of making a type II error) [38]. For all the tests, we obtain values above 0.8, which is the threshold commonly taken as a standard for adequacy for the given sample sizes [38]. Therefore, we reject the null hypothesis and hence conclude that there are significant differences between the two groups...

- D7: *Use appropriate levels of blinding*. In our study, the students knew the parser generation tool they were using. They could have spoken to one another and caused expectations about the utilization of a different tool (ANTLR), not used in the previous years. Thus, we have added a new discussion about this topic in the Threats to Validity section. This is the new paragraph:

  In an empirical research study where users utilize two different tools, the participants are aware of the tool they are using, so a blind experiment is hardly feasible [24]. Therefore, the student's expectations about the use of ANTLR may have represented a psychological factor that might have influenced their opinion of that tool. Although the lecturers did not compare ANTLR with Lex/Yacc, the students may have known they were using different tools because they could have spoken one to another. However, the measurements that are not based on student's opinion (completion percentages, work time, and evaluation data) seem to back up the answers that the students gave in the questionnaires.

- D8: *If you cannot avoid evaluating your own work, then make explicit any vested interests (including your sources of support) and report what you have done to minimize bias*. This guideline is not applicable in our study, since neither ANTLR nor Lex/Yacc are our own work.

- D9: *Avoid the use of controls unless you are sure the control situation can be unambiguously defined*. Our work compares two well-known parser generation tools, one against the other.

- D10: *Fully define all treatments (interventions)*. The article describes the treatments in two main parts. First, Table 3 depicts the work to be done by the students in each lab. Then, the features of the language to be implemented are described in the following text:

  … Labs (Table 3) follow a project-based learning approach, and they are mainly aimed at implementing a compiler of a medium-complexity imperative programming language. That language provides integer, real and character built-in types, arrays, structs, functions, global and local variables, arithmetical, logical and comparison expressions, literals, identifiers, and conditional and iterative control flow statements [25]. The compiler is implemented in the Java programming language.

- D11: *Justify the choice of outcome measures in terms of their relevance to the objectives of the empirical study.* The Evaluation section in the paper justifies the choice of measures in terms of the objectives of our study. First, we indicate we want to analyze the influence of the tools in students' performance:

  We analyze the influence of Lex/Yacc and ANTLR generators in students' performance (students are the experimental units) with different methods…

  Then, we describe the different methods to measure their performance: calculating the completion levels for each lab; recording the number of additional autonomous hours to finish students' work; processing their grades in the lab and theory exams; and comparing their marks with previous years.

  We also ask for their opinion about the tool used, after each lab:

  To gather the student's opinion about the lexer and parser generators used, we asked them to fill in the anonymous questionnaire shown in Table 4, published online with Google Forms. They filled in the questionnaire after the labs where the generators were first used (labs 4 and 5), and after implementing the first part of the language (lab 7) and the whole compiler (lab 15). Questions 4 and 5 were only asked in lab 15,

since students had yet not implemented the semantic analysis and code generation modules in labs 4, 5, and 7…

- Topic DC: Conduct of the experiment and data collection.
  - DC1: *Define all software measures fully, including the entity, attribute, unit and counting rules.* One of the measures that we had not defined clearly in the previous version of the article is the completion level of students' work after each lab. Thus, we have added the following text to better describe how we compute that percentage:

    … First, we measure the completion level of students' work after each lab focused on the use of lexer and parser generators (i.e., labs 4 to 6 in Table 3). Such completion level is measured with a percentage indicating to what extent all the functional requirements of each lab have been met by the student. To this aim, we define a rubric and the lab instructor annotates the completion percentage for each student, after the lab. We perform the same analysis when we review their work after the first part of the course (lab 7) and for the whole compiler (lab 15).
  - DC2: *For subjective measures, present a measure of interrater agreement, such as the kappa statistic or the intraclass correlation coefficient for continuous measures.* This is the answer to the reviewer's Comment 2. As mentioned, we have measured the Krippendorff's alpha coefficient and included the results in the new version of the article.
  - DC3: *Describe any quality control method used to ensure completeness and accuracy of data collection.* We perform the following quality control methods: for all the measurements, we identify and treat outliers (see guideline A3), we compute the Cronbach's α coefficient to measure the internal consistency of our questionnaires (Evaluation section), and we check for normality when t-tests are applied (Evaluation).
  - DC4: *For surveys, monitor and report the response rate and discuss the representativeness of the responses and the impact of nonresponse.* We had not stated the exact number of students attending each lab and answering each questionnaire. Therefore, we have extended the new version of the article to include that information. Table 5 now indicates the number of students attending the labs where we perform measurements. Likewise, a new column has been added to Table 7, indicating the number of students that answered the questionnaire. The greatest difference between the number of students in a group and those attending the labs or filling in the survey is 7.6% (Lex/Yacc group, questionnaire of the last lab). For the initial labs, almost 100% of the students attended the labs and filled in the questionnaire. The lowest response rates were obtained in the last lab (4.3% for attendance and 7.6% for the survey), and the main reason is that those students had quit the course.
  - DC5: *For observational studies and experiments, record data about subjects who drop out from the studies.* The students who dropped out from the course were 4.3% for the Lex/Yacc group and 2.2% for ANTLR. These low percentages do not seem to represent an impact on the results, since all the values obtained for the power of the tests with those sizes were greater than 0.8 (see guideline D6). Moreover, the lab work is individual, so dropping-out students do not affect the work of the others.
  - DC6: *For observational studies and experiments, record data about other performance measures that may be affected by the treatment, even if they are not the main focus of the study.* We do not identify any other performance measures that may be affected by our study. The main variable is students' performance and it is analyzed in the article.

- Topic A: Analysis.
  - A1: *Specify any procedures used to control for multiple testing*. We do not undertake multiple testing in our work.
  - A2: *Consider using blind analysis*. All the analyses done are aimed at analyzing the differences between two groups, so blind analysis is not applicable.
  - A3: *Perform sensitivity analyses*. We searched for outliers in the numerical values of completion rates (Tables 5 and 6) and the number of additional hours to finish the labs (Table 6). All the values were within the Q_3-1.5*IQR and Q_3+1.5*IQR (Tukey's rule, k=1.5). It is worth noting that we did not consider the students who retook the course. This piece of information was not originally in our article, so we have added it to the Context section:

    … Retakers were not included in our study, because they have previous knowledge about the course and experience in the utilization of Lex/Yacc (the tool used in the previous years).
  - A4: *Ensure that the data do not violate the assumptions of the tests used on them*. To apply all the t-tests we performed in our analysis, we checked that it follows a normal distribution computing the Shapiro-Wilk test. In all the tests, p-values were greater than 0.01. This is the text in the article that mentions that:

    … Since the two groups are independent and students' marks are normally distributed (Shapiro-Wilk test [35], p-value>0.1 for all the distributions), we apply an unpaired two-tailed t-test (α=0.05) [36]…
  - A5: *Apply appropriate quality control procedures to verify your results*. We undertook exploratory data analyses and visualization before undertaking our analyses.
- Topic P: Presentation of results.
  - P1: *Describe or cite a reference for all statistical procedures used*. All the statistical procedures we used have been cited. Some of them have also been briefly described. In our new version of the article, we have added references to the Shapiro-Wilk normality test and to the Student's t-test, which had not been included originally.
  - P2: *Report the statistical package used*. We had not reported that in the article, so the following sentence has been included at the end of the Methods section:

    All the statistical computations were performed with IBM SPSS Statistics 27.0.1…
  - P3: *Present quantitative results as well as significance levels. Quantitative results should show the magnitude of effects and the confidence limits*. The only quantitative result in the guideline that we had not included is the t statistic. Thus, we have added a new column to Table 6 to include the t statistic of the t-tests.
  - P4: *Present the raw data whenever possible. Otherwise, confirm that they are available for confidential review by the reviewers and independent auditors*. We have taken the raw data gathered from all the experiments described in the article and uploaded them to [37]. The following text has been added to the last paragraph of the Evaluation section:

    … The raw data obtained from conducting all the experiments are available for download from [37].
  - P5: *Provide appropriate descriptive statistics*. This guideline was followed in the interpretation of student's opinion. As described by Boone *et al.* [39] the Likert scale data obtained by summing the values of four or more Likert-type items can be used to compare values at the interval measurement scale [39]. That is precisely what we

do in Figure 2: we computed the combined (sum) Likert scale values for Q1-Q5 and then perform t-test to compare the two groups.

- P6: *Make appropriate use of graphics*. All the guidelines for graphical representations described in P6 have been followed. We have also respected the principles of "The Visual Display of Quantitative Information" by Edward Tufte.

- Topic I: Interpretation of results.
  - I1: *Define the population to which inferential statistics and predictive models apply*. We have thoroughly described the population (guideline D1) and its context (guideline C1). Regarding the distribution of the attribute values of the population, we have first checked for their normality before applying the statistical tests.
  - I2: *Differentiate between statistical significance and practical importance*. For all the hypothesis tests undertaken to compare the values of the ANTLR and Lex/Yacc groups, we compute the p-value as a measure of significance. Afterwards, when a significance level is achieved, we calculate the difference between the mean values of both distributions and discuss the practical importance of that difference.
  - I3: *Define the type of study*. This guideline cites the study of Zelkowitz and Wallace [42] as a valid reference for classifying empirical research work in software engineering, where 12 types of experimental methods are identified. According to their study, our work is a controlled experiment within a synthetic environment. We now define the type of study in the following text included in the Threats to Validity section:

    Likewise, the study was conducted with Software Engineering students with Java programming skills, all of them beginners of the parser generation tools (students retaking the course were not considered). According to Zelkowitz and Wallace, this kind of validation method is a controlled experiment within a synthetic environment, where two technologies are compared in a laboratory setting [42]...
  - I4: *Specify any limitations of the study*. We have the Threats to Validity section in the article to specify all the limitations of our study.

Comment 3: In addition one typo is found in line 48 on page 3, "the related related"?

Response 3: Our apologies. We have replaced the mistaken "related related" text with "related work".

Finally, we would like to thank the anonymous reviewer for all his/her suggestions and comments to help us improve the article.

Reviewer #6:

Comment 1: Parsers are used various software development scenarios, two of which are Lex/Yacc and ANTLR. Surprisingly, though ANTLR provides more features, Lex/Yacc is used more in university courses settings. Different approaches exist for qualitative comparisons of the features provided by ANTLR and Lex/Yacc. However, no empirical study evaluates features such as language implementor productivity and tool simplicity, intuitiveness, and maintainability. This paper fills this gap with an empirical experiment with undergraduate students of a Software Engineering degree in a compiler design and implementation course. The students are divided into two random groups, implementing the same language with a different parser generator. The performance of both groups are statistically compared. The results show that use of ANTLR increased pass rates and exam attendance percentages.

It is amusing that ANTLR is used less than Lex/Yacc in university courses settings. This study shows the ANTLR is only used in one of fourteen top universities for compiler design and implementation course. This suggests the current status quo needs change as ANTLR has more features and is more accepted by the students in the empirical study, e.g, the students satisfaction with ANTLR, the increased passing rates and better exam attendance.

The authors comprehensively compared Lex/Yacc and ANTLR features such as parsing strategy. As with the results, the authors carefully designed a questionnaire with multiple questions for the students to evaluate the difference of the tools. This paper also contains a variety of results regarding such as the percentage of students who completed different activities related to lexer and parser implementations.

A minor point and a suggestion may be in table 1, the authors can also add top universities from USNEWS ranking.

Response 1: As suggested by the reviewer, we have included the US News ranking "best global universities for computer science". There are two universities in this raking that were not included in Table 1: Peking University and The Chinese University of Hong Kong. We have added both in the new version of the article.

This is the new Table 1:

Table 1. Parser generation tools used in the compiler courses of the top 10 universities according to Shanghai Consultancy [6], Quacquarelli Symonds [7], Times Higher Education [8], and US News [9] rankings.

University	Course name	Parser generator used
Carnegie Mellon University	Compiler design	ML-Lex/ML-Yacc
ETH Zurich	Compiler design	OCamlLex/Menhir
Harvard University	Compilers	OCamlLex/Menhir
Imperial College London	Compilers	Lex/Yacc
Massachusetts Institute of Technology	Computer language engineering	ANTLR
Nanyang Technological University	Compiler technology of programming languages	Lex/Yacc
National University of Singapore	Compiler design	JFlex/CUP
Peking University	Principles of Programming Language Design	OCamlLex/Menhir
Stanford University	Compilers	Flex/Bison
Swiss Federal Institute of Technology in Lausanne	Computer language processing	Manual implementation with a LL(1) grammar
The Chinese University of Hong Kong	Compiler Construction	JavaCC
Tsinghua University	Principles of assembly and compilation	Lex/Yacc
University of California, Berkeley	Programming languages and compilers	JFlex/CUP
University of Cambridge	Compiler construction	OCamlLex/OCamlYacc
University of Oxford	Compilers	OCamlLex/OCamlYacc
University of Toronto	Compiler and interpreters	Flex/Bison

Only 16 universities appear in Table 1 because some of them are within the top 10 positions in more than one ranking. If information about the parser generator is not provided, the university is not included. Universities are ordered alphabetically.

The text of the article has been changed accordingly to reflect the changes in Table 1.

Comment 2: Overall, this paper represents an original research study, in which the data supports its conclusions. It is also well-written in English. And to the best of our knowledge, it is not published elsewhere before.

Response 2: We like to sincerely thank the reviewer for his/her time and effort to read and review our article, and for his/her positive comments.
